# Concordance between DSM-IV and DSM-5 criteria for delirium diagnosis in a pooled database of 768 prospectively evaluated patients using the delirium rating scale-revised-98

**DOI:** 10.1186/s12916-014-0164-8

**Published:** 2014-09-30

**Authors:** David J Meagher, Alessandro Morandi, Sharon K Inouye, Wes Ely, Dimitrios Adamis, Alasdair J Maclullich, James L Rudolph, Karin Neufeld, Maeve Leonard, Giuseppe Bellelli, Daniel Davis, Andrew Teodorczuk, Stefan Kreisel, Christine Thomas, Wolfgang Hasemann, Suzanne Timmons, Niamh O’Regan, Sandeep Grover, Faiza Jabbar, Walter Cullen, Colum Dunne, Barbara Kamholz, Barbara C Van Munster, Sophia E De Rooij, Jos De Jonghe, Paula T Trzepacz

**Affiliations:** Graduate-entry Medical School, University of Limerick, Limerick, Ireland; Cognitive Impairment Research Group, Centre for Interventions in Infection, Inflammation & Immunity (4i), Graduate Entry Medical School, University of Limerick, Limerick, Ireland; Department of Psychiatry, University Hospital Limerick, Limerick, Ireland; Rehabilitation and Aged Care Unit Hospital Ancelle, Cremona, Italy; Geriatric Research Group, Brescia, Italy; Aging Brain Center, Institute for Aging Research, Hebrew SeniorLife, Boston, MA USA; Division of Gerontology, Department of Medicine, Beth Israel Deaconess Medical Center, Harvard Medical School, Boston, MA USA; Vanderbilt University Medical Center, Pulmonary and Critical Care, Nashville, TN USA; Tennessee Valley VA Geriatric Research Education Clinical Center (GRECC), Nashville, TN USA; Research and Academic Institute of Athens, Greece, 27 Themistokleous Street and Akadimias, Athens, 106 77 Greece; Sligo Mental Health Services, Ballytivan, Sligo, Ireland; Edinburgh Delirium Research Group, Geriatric Medicine, Division of Health Sciences, School of Clinical Sciences, University of Edinburgh, Edinburgh, Scotland UK; Centre for Cognitive Ageing and Cognitive Epidemiology, University of Edinburgh, Edinburgh, Scotland UK; VA Boston Healthcare System, Geriatric Research, Education, and Clinical Center and Division of Geriatrics and Palliative Care, Boston, MA USA; Brigham and Women’s Hospital, Division of Aging, Boston, MA USA; Harvard Medical School, Boston, MA USA; Department of Psychiatry and Behavioral Sciences, Johns Hopkins University School of Medicine, Baltimore, MD USA; University of Milano Bicocca and Geriatric Medicine, San Gerardo Hospital, Monza, Italy; Department of Public Health and Primary Care, University of Cambridge, Cambridge, UK; Institute for Ageing and Health, Campus for Vitality, Newcastle University, Newcastle upon Tyne, UK; Department of Psychiatry and Psychotherapy, Bethel EvangelischesKrankenhaus, Bielefeld, Germany; Department of Psychiatry and Psychotherapy of the Aged, Centre of Mental Health, Klinikum Stuttgart, Germany; Practice Development in Nursing, University Hospital Basel, Basel, Switzerland; Centre for Gerontology and Rehabilitation, School of Medicine, University College Cork, Cork, Ireland; Department of Psychiatry, Postgraduate Institute of Medical Education & Research, Chandigarh, 160012 India; Psychiatry for Later Life Service, University College Hospital, Galway, Ireland; Mental Health Services, San Francisco VA Medical Center, San Francisco, CA USA; Department of Internal Medicine, Geriatrics Section, Academic Medical Center, University of Amsterdam, Amsterdam, The Netherlands; Department of Geriatrics, Gelre Hospitals, Apeldoorn, The Netherlands; Department of Geriatric Medicine, Medical Center Alkmaar, Alkmaar, The Netherlands; Lilly Research Laboratories, Indianapolis, IN USA; University of Mississippi Medical School, Jackson, MS USA; Tufts University School of Medicine, Boston, MA USA; Indiana University School of Medicine, Indianapolis, IN USA

**Keywords:** Delirium, Classification, Diagnosis, Cognition, Neurocognitive disorders, Dementia

## Abstract

**Background:**

The *Diagnostic and Statistical Manual* fifth edition (DSM-5) provides new criteria for delirium diagnosis. We examined delirium diagnosis using these new criteria compared with the *Diagnostic and Statistical Manual* fourth edition (DSM-IV) in a large dataset of patients assessed for delirium and related presentations.

**Methods:**

Patient data (n = 768) from six prospectively collected cohorts, clinically assessed using DSM-IV and the Delirium Rating Scale-Revised-98 (DRS-R98), were pooled. *Post hoc* application of DRS-R98 item scores were used to rate DSM-5 criteria. ‘Strict’ and ‘relaxed’ DSM-5 criteria to ascertain delirium were compared to rates determined by DSM-IV.

**Results:**

Using DSM-IV by clinical assessment, delirium was found in 510/768 patients (66%). Strict DSM-5 criteria categorized 158 as delirious including 155 (30%) with DSM-IV delirium, whereas relaxed DSM-5 criteria identified 466 as delirious, including 455 (89%) diagnosed by DSM-IV (*P* <0.001). The concordance between the different diagnostic methods was: 53% (ĸ = 0.22) between DSM-IV and the strict DSM-5, 91% (ĸ = 0.82) between the DSM-IV and relaxed DSM-5 criteria and 60% (ĸ = 0.29) between the strict versus relaxed DSM-5 criteria. Only 155 cases were identified as delirium by all three approaches. The 55 (11%) patients with DSM-IV delirium who were not rated as delirious by relaxed criteria had lower mean DRS-R98 total scores than those rated as delirious (13.7 ± 3.9 versus 23.7 ± 6.0; *P* <0.001). Conversely, mean DRS-R98 score (21.1 ± 6.4) for the 70% not rated as delirious by strict DSM-5 criteria was consistent with suggested cutoff scores for full syndromal delirium. Only 11 cases met DSM-5 criteria that were not deemed to have DSM-IV delirium.

**Conclusions:**

The concordance between DSM-IV and the new DSM-5 delirium criteria varies considerably depending on the interpretation of criteria. Overly-strict adherence for some new text details in DSM-5 criteria would reduce the number of delirium cases diagnosed; however, a more ‘relaxed’ approach renders DSM-5 criteria comparable to DSM-IV with minimal impact on their actual application and is thus recommended.

## Background

Delirium is a complex neuropsychiatric syndrome that is common across healthcare settings, occurring in 29% to 64% of medical in-patients [1,2] with even higher rates among patients in intensive and palliative care settings [3]. It is independently associated with a range of adverse outcomes that include elevated risk of dementia and mortality [4,5]. However, delirium is often misdiagnosed and under detected in real-world practice [6–8] such that clear and concise diagnostic criteria are fundamental to improving detection and management.

The advent of clear diagnostic criteria for delirium in the *Diagnostic and Statistical Manual* third edition (DSM-III) and subsequent *Diagnostic and Statistical Manual* third edition revised (DSM-IIIR) and the *Diagnostic and Statistical Manual* fourth edition (DSM-IV) [9–11] versions has supported considerable growth in research activity in the field of delirium [12]. The DSM-IV criteria provide a highly inclusive description of delirium that has become the preferred diagnostic criteria for both clinicians and researchers [13]. However, the essential criteria have been progressively abbreviated [14] and studies indicate considerable disparity in delirium detection when applying these different DSM versions and the *International Classification of Diseases* – Tenth Edition (ICD-10) [15–19].

The fifth revision of the *Diagnostic and Statistical Manual of Mental Disorders* (DSM-5) [20] provides an opportunity to consolidate the strengths of the DSM-IV delirium description while incorporating findings from interim research. Although no major changes from DSM-IV were made to the core elements of DSM-5 criteria for delirium, there are some differences in content and wording of the criteria (Table 1) that may impact upon the alignment between DSM-5 and previous criteria. For example, the removal of the term ‘consciousness’, and the focus on reduced awareness and inattention might substantially narrow the inclusiveness of the criteria, depending on how strictly this term is interpreted. The application of DSM-5 criteria could impact substantially upon both clinical care and research case identification, such that it is important to understand how they compare in the same patient population.

**Table 1:** **A comparison of DSM-IV and DSM-5 criteria for delirium**

**DSM-5**	**DSM-IV**	**Comments**
A. A disturbance in attention (i.e., reduced ability to direct, focus, sustain, and shift attention) and awareness (reduced orientation to the environment).	A. Disturbance of consciousness (i.e., reduced clarity of awareness of the environment) with reduced ability to focus, sustain or shift attention.	The cardinal criterion for DSM-5 and DSM-IV includes both inattention and reduced awareness of the environment. Although attention and awareness are important components of normal consciousness, they do not fully represent it. The suggestion that orientation to the environment indicates awareness is new to DSM-5.
B. The disturbance develops over a short period of time (usually hours to a few days), represents a change from baseline attention and awareness, and tends to fluctuate in severity during the course of a day.	C. The disturbance develops over a short period of time (usually hours to days) and tends to fluctuate during the course of the day	Both capture acuity of onset and fluctuation of severity.
		Change from baseline is noted only in DSM-5 as this relates to attention and awareness.
C. An additional disturbance in cognition (e.g. memory deficit, disorientation, language, visuospatial ability, or perception).	B. A change in cognition or the development of a perceptual disturbance that is not better accounted for by a pre-existing, established or evolving dementia.	DSM-5 lists examples of other affected cognitive domains with perception. Change from baseline for other cognitive domains is noted in DSM-IV.
D. The disturbances in Criteria A and C are not better explained by a pre-existing, established or evolving neurocognitive disorder and do not occur in the context of a severely reduced level of arousal, such as coma.		Unlike DSM-IV, DSM-5 criteria specifically excludes coma from being labelled as delirium but suggests that where reduced arousal impairs ability to engage with cognitive testing that this can be deemed evidence of severe inattention. Both exclude dementia as the primary cause of the disturbance while DSM-5 more broadly includes other neurocognitive disorders besides dementia.
E. There is evidence from the history, physical examination or laboratory findings that the disturbance is a direct physiological consequence of another medical condition, substance intoxication or withdrawal, or exposure to a toxin, or is due to multiple etiologies.	D. There is evidence from the history, physical examination or laboratory findings that the disturbance is caused by the direct physiological consequences of a general medical condition.	DSM-5 has a broader list of etiological types.

Note: Adapted to allow direct item comparison from DSM-IV (American Psychiatric Association, 1994) and DSM-5 (American Psychiatric Association, 2013). DSM-IV, *Diagnostic and Statistical Manual* fourth edition; DSM-5, *Diagnostic and Statistical Manual* fifth edition.

Moreover, although the gold standard for delirium identification is considered a clinical diagnosis according to DSM-IV criteria, there is widespread recognition among ‘deliriumologists’ that there is no consensus as to how this should actually be determined in practice. Against this background, and considering the new DSM-5 criteria, we explored how the criteria can be applied when the individual elements are assessed in a systematic and operationalized way, thus allowing for various interpretations of the new criteria to be examined. In order to explore how DSM-5 criteria might differ from those of DSM-IV, we examined a pooled dataset derived from previous prospective phenomenological research exploring delirium in a variety of clinical populations and research sites using standardized assessments.

The aims of this study were: (1) to analyze retrospectively the pooled database to compare features of delirium cohorts identified by the originally applied DSM-IV criteria (identified by the gold standard of a detailed clinical assessment) as well as those identified by applying scores from the Delirium Rating Scale-Revised-98 (DRS-R98) [21] items relevant to criteria for a *post hoc* application of *strict* and *relaxed* interpretations of DSM-5 criteria; (2) to examine whether different interpretations of DSM-5 criteria impact significantly upon delirium identification rates by exploring concordance across these three groups. We thus sought to understand to what degree DSM-IV and DSM-5 were concordant and how the DSM-5 criteria can be best applied to allow for inclusiveness and that the gap between rates of diagnosis by the different systems is not excessively wide; and (3) to examine how DSM-IV and the different interpretations of DSM-5 differ in terms of delirium phenomenology.

## Methods

### Samples and study design

The pooled dataset derives from six related phenomenological studies exploring the neuropsychiatric profile of patients with delirium and related conditions from a variety of clinical settings that were conducted under the umbrella of the Cognitive Impairment Research Group (CIRG) at the University of Limerick in Ireland. In all studies phenomenology, demographic and treatment data were assessed in a standardized manner by raters (DM, ML, FJ KC, ST, JF) who were all trained by an expert in the use of the DRS-R98 (DM) using the DRS-R98 Administration Manual [22].

The analyses reported used cross-sectional assessments involving all available data and were conducted on the first day of delirium assessment. The dataset consists of 768 patients, 510 (65%) of whom received an original diagnosis of delirium from a trained psychiatrist using DSM-IV criteria, with 258 (35%) non-delirium patients from the same clinical settings, the majority of whom had been referred for assessment of possible delirium to consultation-liaison psychiatry services. DSM-IV criteria for delirium [11] were rated according to all available data for each patient, including clinical interview and assessments, consultation with nursing staff, medical records and collateral history from caregivers where available. Patients who were unable to co-operate with assessments (for example, due to severely reduced arousal where it was not possible for them to meaningfully engage in assessments even for brief periods) were not included in these studies.

Studies included in the pooled database analysis are described in Table 2. For three studies [2,23,24], cases (n = 402, 525 of total; 255 cases of delirium, 50% of DSM-IV cases) were identified using screening with the Confusion Assessment Method (CAM) [25] after formal training to increase accuracy. The remainder of the studies [26,27] evaluated consecutively referred cases. Four of six studies included non-delirium cases.

**Table 2:** **Studies included in the pooled dataset**

**Study**	**Population**	**Number**	**Study design**	**Age (mean ± SD)**	**Male number (%)**	**CAM screening**	**Dementia assessment**
Meagher *et al*. [23]	Palliative care	100 delirium	Cross sectional	70.1 ± 11.5	50	Yes	Clinical diagnosis
	Limerick, Ireland						
Meagher *et al*. [24]	Palliative Care	100 delirium	Longitudinal	70.2 ± 10.5	51	Yes	Clinical diagnosis
				69.6 ± 11.6	49		
	Limerick, Ireland						
		69 nondelirium					
Jabbar *et al*. [26]	Psychogeriatric C/L referrals	80 delirium	Cross-sectional	79.3 ± 7.7	49	No	Clinical diagnosis
	Galway and Limerick, Ireland						
Grover *et al*. [27]	C/L Psychiatry referrals	100 delirium	Cross-sectional	44.4 ± 19.4	78	No	Clinical diagnosis
				43.9 ± 14.6	69		
	Chandigarh, India	60 nondelirium					
Ryan *et al*. [2]	General hospital inpatients	55 delirium	Cross-sectional	76.0 ± 16.6	50	Yes	IQCODE
		78 nondelirium					
				67.1 ± 18.8	50		
	Cork, Ireland						
Meagher *et al*. (unpublished)	Psychogeriatric C/L referrals	75 delirium	Cross-sectional	80.1 ± 8.3	46	No	IQCODE
		51 nondelirium					
	Limerick, Ireland						
				79.0 ± 17.2	41		

*CAM* Confusion Assessment Method, *IQCODE* Informant Questionnaire on Cognitive Decline in the Elderly.

For each of the groups studied, the presence of prior cognitive impairment or dementia was attributed if there was evidence of any of the following: (1) a documented history of dementia in clinical case notes; (2) recognized diagnosis of dementia evident by collateral history from a reliable source; (3) a history of prior cognitive impairment of at least six months duration; or (4) Short- Informant Questionnaire on Cognitive Decline in the Elderly (IQCODE) score [28] of >3.5 (conducted in Ryan *et al*., [2]; Meagher *et al*., unpublished samples). Any cases of uncertainty were resolved by the delirium research and primary medical teams, with a regular CIRG consensus meeting to facilitate diagnosis in more complex cases.

### Procedures

The DRS-R98 [21] is a widely used instrument for measuring the symptom profile in delirium that can be used both as a diagnostic and severity assessment tool. It is a 16-item clinician-rated scale (DRS-R98 Total scale) with 13 severity items (Severity scale) and 3 diagnostic items. All items are anchored by text descriptions which guide rating along a continuum from normal (0), abnormal/present but possibly within normal limits of behavior (1), present and abnormal (2), present and severe in intensity (3). A cutoff score ≥18 on the total scale is consistent with a diagnosis of delirium. It is designed to rate symptoms over the previous 24 hours. The DRS-R98 has high inter-rater reliability and is both sensitive (91% to 100%) and specific (85% to 100%) for distinguishing delirium in populations with mixed neuropsychiatric presentations including dementia, depression and schizophrenia [21,29]. Throughout this paper, DRS-R98 refers to the Total scale score unless otherwise specified.

The CIRG used a standardized approach to clinical rating of the DRS-R98 based upon the DRS-R98 Administration Manual [22] which utilizes both objective testing and subjective interviewer-based judgments for rating item severities, where particular tests and interview questions are used as probes for symptoms. In order to standardize DRS-R98 rating performance across CIRG studies, we developed and utilized training procedures that included a workshop and video case vignettes. Additionally, for this report, relevant DRS-R98 items were selected to serve as content proxies for the presence of DSM-5 criteria to generate the *post hoc* determination of DSM-5 criteria. DSM-5 was defined in two ways: strict criteria (for example, requiring all criteria in their most explicit forms) versus relaxed criteria, where features were included in all possible forms.

The strict and relaxed DSM-5 criteria for the *post hoc* proxy qualification of the presence of DSM-5 delirium criteria using DRS-R98 items areas are shown in Table 3. The two interpretations differ principally in relation to criteria A and B. For criterion A, the strict criterion required evidence of impaired attention as well as impaired awareness evidenced by ‘impaired orientation to the environment’ required to have a documented impairment of orientation to time, place or person as tested in formal cognitive assessment for the DRS-R98 Orientation item. The relaxed interpretation did not require the latter but focused upon disturbed attention by applying only DRS-R98 evidence of inattention. For criterion B, the strict interpretation required both acute onset and fluctuating symptom pattern, while the relaxed interpretation required either acute/subacute onset or fluctuation symptom course.

**Table 3:** **Procedures for assessing DRS-R98 items relevant to DSM-5 criteria for delirium**

**DSM-5 criteria**		**Application of criteria**	
	***Post hoc*** **rating**	**Strict**	**Relaxed**
**Criterion A**			
A disturbance in attention	*DRS-R98 item 10 Attention score ≥1*	●	●
and awareness with reduced orientation to the environment	*DRS-R98 item 9 Orientation score ≥1*	●	
**Criterion B**			
The disturbance develops over a short period of time (usually hours to a few days)	*DRS-R98 item 14 Temporal Onset ≥1*	●	*either*
represents a change from baseline attention and awareness, and tends to fluctuate in severity during the course of a day	*DRS-R98 item 15 Fluctuation score ≥1*	●	
**Criterion C**			
An additional disturbance in cognition (e.g. memory deficit, disorientation, language, visuospatial ability, or perception	*Any of the following: score of ≥1 on DRS-R98 item 11 Short term memory, item 9 Orientation, and Score of ≥2 on DRS-R98 item 5 Language, item 13 Visuospatial, item 2 Perceptual disturbance.*	●	●
**Criterion D**			
The disturbances in Criteria A and C are not better explained by a pre-existing, established or evolving neurocognitive disorder and do not occur in the context of a severely reduced level of arousal, such as coma.	*Where dementia is present, a total DRS-R98 score of ≥18 denoted presence of comorbid delirium*	●	●
**Criterion E**			
There is evidence from the history, physical examination or laboratory findings that the disturbance is a direct physiological consequence of another medical condition, substance intoxication or withdrawal, or exposure to a toxin, or is due to multiple etiologies	*DRS-R98 item 16 Etiology score ≥1*	●	●

*DRS-98* Delirium Rating Scale-Revised-98, *DSM-5* *Diagnostic and Statistical Manual* fifth edition.

### Informed consent

Similar bioethical procedures were used for all patient groups. The procedures and rationale for assessments were explained to all patients but because many had delirium at study entry it was presumed that most would not be capable of giving informed written consent. At each site, local ethics committees approved an approach whereby patient verbal assent was augmented with proxy consent from next of kin (where possible) or a responsible caregiver. This is in accordance with the Helsinki Guidelines for medical research involving human subjects [30]. These assessment procedures did not have any identified significant risks but the patient or family was informed that they could withdraw participation at any stage.

### Statistical analysis

Statistical analysis was conducted using the SPSS v19.0 package for windows. Delirium versus non-delirium groups by each of the three diagnostic systems were compared for continuous variables (age, total DRS-R98 scores) using independent t-tests and for non-normal data (for example, item frequencies, comorbid dementia frequency) using Chi-squared tests. The agreement between different criteria was assessed using Cohen’s kappa.

The whole population was subdivided into three clinical groups each comprised of two studies - palliative care (n = 269), general hospital inpatients (n = 293) and psychiatry for later life patients (n = 206) - to examine how the patterns of concordance compared across clinical populations.

DSM-5 criteria were populated using DRS-R98 item scores (see Table 4) of at least one point to determine the presence of that symptom in meeting a criterion, according to the approach for either strict or relaxed interpretations (see above) as applied by the first author (DM). To standardize our methodology of this *post hoc* method using DRS-R98 item scores as proxies for clinical interview, we first evaluated the concordance between actual DSM-IV delirium caseness with *post hoc* DRS-R98 proxy DSM-IV diagnosis; because we found high concordance (89%; ĸ = 0.76.) [31], we were confident in the output for comparisons between DSM-IV actual and DSM-5 proxy in our report.

**Table 4:** **Sensitivity, specificity and predictive accuracy of DSM-5 strict and relaxed criteria for DSM-IV delirium**

**Detection accuracy measure**	**Strict criteria**	**Relaxed criteria**
number/Number (%) (95% CI)		
**Sensitivity**	155/510 (30) (26 to 35)	455/510 (89) (86 to 92)
**Specificity**	255/258 (99) (97 to 99)	247/258 (96) (93 to 98)
**Positive Predictive Value**	155/158 (98) (95 to 99)	455/466 (98) (96 to 99)
**Negative Predictive Value**	255/610 (42) (38 to 46)	247/302 (82) (77 to 86)

*CI* confidence interval, *DSM-IV* *Diagnostic and Statistical Manual* fourth edition, *DSM-5* *Diagnostic and Statistical Manual* fifth edition.

## Results

### Identification of delirium by diagnostic criteria

The pooled dataset contained 768 patients: 510 (65%) with DSM-IV delirium as established by virtue of a detailed clinical assessment of all available information and 258 (35%) without delirium. The demographic and clinical characteristics of these groups are compared in Table 5. Application of the DSM-5 criteria identified 158 cases (strict criteria) and 466 cases (relaxed criteria) of delirium (*P* <0.001). The three approaches to diagnosis produce largely similar groups but the differences with respect to the age profile and comorbid dementia rate for the DSM-IV versus the DSM-5 relaxed interpretation highlight that a higher proportion of the cases of DSM-IV delirium that were not included within DSM-5 relaxed criteria have evidence of dementia (*P* <0.001) and were significantly older (*P* <0.01).

**Table 5:** **Clinical and demographic characteristics of the whole population and subgroups as determined by DSM-IV and DSM-5 delirium criteria**

	**Whole group**	**DSM-IV criteria**		**Strict DSM-5 criteria**		**Relaxed DSM-5 criteria**	
		**Delirium**	**No delirium**	**Delirium**	**No delirium**	**Delirium**	**No delirium**
**Number**	768	510	258	158	610	466	302
**Mean age** ^**a**^	67.4 ± 18.7	68.7 ± 18.2	64.6 ± 19.9	70.8 ± 16.9	66.5 ± 19.3	67.9 ± 18.5	67.3 ± 18.2
**Sex (% male)**	49	53	45	50	50	55	40
**Frequency of comorbid dementia (%)** ^**b**^	28	30	25	28	30	24	37
**Mean DRS-R98 total scores** ^**c**^	17.2 ± 9.7	22.6 ± 6.5	6.6 ± 4.9	25.9 ± 5.8	14.9 ± 9.2	23.5 ± 6.2	7.6 ± 5.1

^a^ *P* <0.01 for delirium versus non-delirium groups for DSM-IV and strict DSM-5 criteria; ^b^ *P* <0.001 for delirium versus non-delirium groups for relaxed DSM-5 criteria; ^c^ *P* <0.001 for delirium versus non-delirium groups for all criteria. *DRS-R98* Revised Delirium Rating Scale – 1998 version, *DSM-IV* *Diagnostic and Statistical Manual* fourth edition, *DSM-5* *Diagnostic and Statistical Manual* fifth edition.

**Figure 1:**
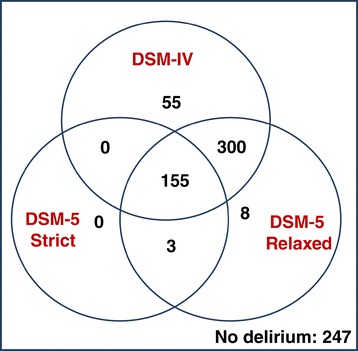
**Overlap between DSM-IV and strict versus relaxed interpretations of DSM-5 delirium criteria for the pooled dataset (n = 768).** Note: Relaxed interpretation of DSM-5 criteria allows for considerable overlap with DSM-IV with respect to delirium diagnosis, while strict interpretation only identified 30% of DSM-IV cases as delirium. DSM-IV, Diagnostic and Statistical Manual fourth edition; DSM-5, *Diagnostic and Statistical Manual* fifth edition.

For the complete cohort (n = 768) the concordance among DSM-IV and the two definitions of DSM-5 is graphed as a Venn diagram in Figure 1 and shown in Table 4 with sensitivity, specificity, positive and negative predictive values for the strict and relaxed DSM-5 criteria in relation to DSM-IV delirium criteria. The concordance between the different diagnostic methods for the whole dataset was 53% (ĸ = 0.22) between DSM-IV and the strict DSM-5, 91% (ĸ = 0.82) between the DSM-IV and relaxed DSM-5 criteria and 60% (ĸ = 0.29) between the strict versus relaxed DSM-5 criteria. Only 155 cases were identified as delirium by all three approaches and 455 were identified by both DSM-IV and relaxed DSM-5. There were three cases of delirium unique to the strict DSM-5 versus DSM-IV group, compared to 355 DSM-IV cases of delirium negative by the strict interpretation of DSM-5. There were 55 delirium cases unique to DSM-IV as compared to either DSM-5 group (characteristics described below). Further, 11 patients met DSM-5 relaxed criteria but not DSM-IV delirium.

We also examined the concordance between the diagnostic methods within different populations. The concordance between DSM-IV and the strict DSM-5 criteria was 48% (k = 0.19) for palliative care patients, 56% (k = 0.16) for general hospital patients and 55% (k = 0.23) for psychiatry for later life patients. For DSM-IV and the relaxed DSM-5 criteria the concordance was 94% (k = 0.85) for palliative care patients, 94% (k = 0.87) for general hospital patients and 86% (k = 0.66) for psychiatry for later life patients. Concordance between the relaxed and strict DSM-5 criteria was 55% (k = 0.25) for palliative care patients, 57% (k = 0.17) for general hospital patients and 69% (k = 0.43) for psychiatry for later life patients.

### Factors underpinning diagnostic differences

We examined the individual features that underpin discordance between systems. For the 355 DSM-IV delirium cases that were negative by strict DSM-5 interpretation, 254 (72%) did not meet the acute and fluctuating criteria, while 83 (23%) lacked evidence of both inattention and disorientation. In comparing DSM-IV cases with (n = 155) and without (n = 355) DSM-5 strict criteria, the latter were significantly older (*P* =0.01) and had significantly higher total DRS-R98 scores (*P* <0.001) due to higher scores for all DRS-R98 individual items except language, short-term memory and visuospatial function. These two groups did not differ significantly in terms of gender or frequency of comorbid dementia.

Of the 149 cases of DSM-IV delirium with comorbid dementia, 39 had DRS-R98 scores under the cutoff of 18 and were deemed non-delirious by both strict and relaxed interpretations.

### Delirium severity and diagnostic concordance

**Figure 2:**
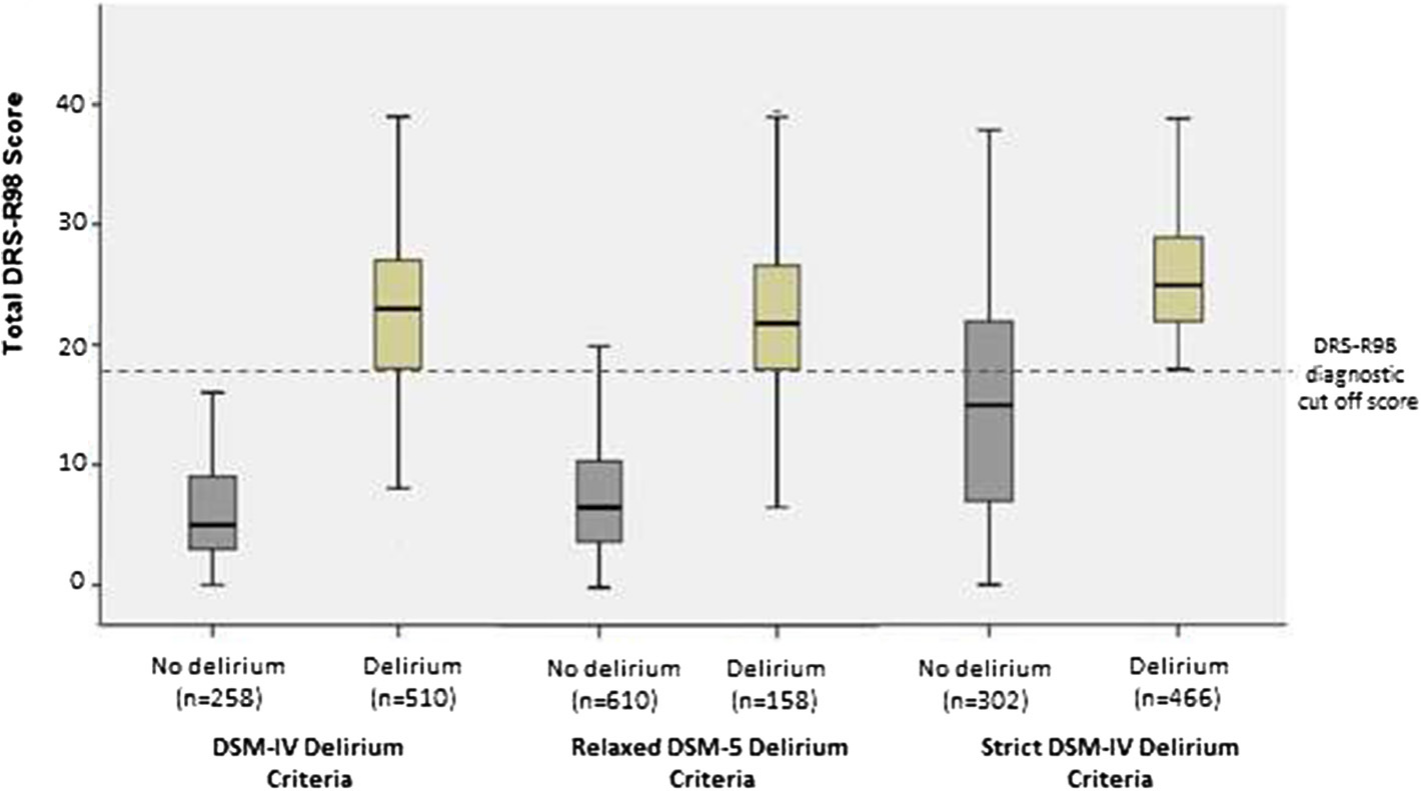
**Total DRS-R98 scale scores for delirium versus non-delirium by DSM-IV and DSM-5 relaxed and strict criteria.** Note: Both DSM-IV and the relaxed interpretation of DSM-5 criteria allow for clear distinction between delirium and no delirium in terms of DRS-R98 scores, but the strict interpretation of DSM-5 excludes many patients with DRS-R98 scores that are consistent with delirium. DSM-IV, *Diagnostic and Statistical Manual* fourth edition; DSM-5, *Diagnostic and Statistical Manual* fifth edition.

The severity of delirium according to DRS-R98 scores for delirious versus non-delirious patients by each of the three diagnostic criteria is shown in Figure 2. Patients with DSM-IV delirium who were excluded (n = 353) by the strict DSM-5 criteria had significantly lower DRS-R98 scores than those who met both DSM-IV and strict DSM-5 criteria (n = 155) (21.1 ± 6.4 versus 25.9 ± 5.9; t = −8.0; df = 508; *P* <0.001), but, of note, both groups had mean DRS-R98 scores indicative of full syndromal delirium. With respect to individual symptoms, this difference was accounted for by significantly greater disturbances in those meeting strict DSM-5 criteria versus those who only met relaxed DSM-5 criteria in the severity of DRS-R98 items for sleep-wake cycle disturbances, perceptual disturbances, delusions, long-term memory, temporal onset of symptoms and physical disorder (all *P* <0.01). Similarly, there was a significant difference between those patients with DSM-IV delirium who met DSM-5 relaxed criteria (n = 455) versus those who did not (n = 55) (23.7 ± 6.0 versus 13.7 ± 3.9; t = −11.9; df = 508; *P* <0.001) but in this case the negative group mean DRS-R98 score was below the DRS-R98 diagnostic cutoff for delirium. These groups differed significantly (*P* < .001) for all of the individual DRS-R98 items except for motor retardation.

## Discussion

The principal purpose of these analyses was to explore the concordance/discordance between DSM-IV and the new DSM-5 criteria for delirium, especially given the differences in wording that could be interpreted differently by different DSM-5 users, thereby leading to potentially widely disparate diagnostic rates with serious implications for patient care and research. We used proxy symptom information captured systematically using a standardized administration of the DRS-R98 to retrospectively generate delirium diagnoses according to two different interpretations of DSM-5 criteria. We then compared the level of concordance between these criteria with each other and with prospectively-determined DSM-IV delirium identified through detailed and comprehensive clinical assessment. This extrapolation was possible because we first tested the proxy method for DSM-IV and found high concordance, supporting the *post hoc* method as valid. We found that a more relaxed interpretation of DSM-5 criteria detected most of the cases of DSM-IV defined delirium, while a strict interpretation excluded more than two-thirds of such cases. There was thus considerable overlap for the actual cases designated as delirious by DSM-IV and the relaxed DSM-5 approach, suggesting that delirium diagnosis will be minimally changed by the use of DSM-5 criteria if the presence of impaired attention is interpreted as sufficient for criterion A, and either acute/subacute onset *or* fluctuation symptom course is used to interpret criterion B.

Studies exploring the concordance between DSM IIIR, DSM-IV and ICD-10 [32] diagnostic systems in elderly hospitalized patients indicate that DSM-IV provides a highly inclusive definition of delirium that includes a substantial number of patients who do not have delirium as defined by DSM-IIIR or ICD-10 [15–19]. The inclusion of disorganized thinking as a criterion in DSM-IIIR [33] and the wide range of mandatory features in ICD-10 are key factors that underpin this finding. Other work suggests that DSM-IV includes many cases that are of subsyndromal severity according to DRS-R98 cut-off scores, although the distinction between subsyndromal versus ‘mild’ delirium can be challenging, especially when dimensional approaches are employed [24,34]. Subsyndromal delirium is addressed under ‘attenuated delirium syndrome’ in DSM-5.

Our work examines how different interpretations can strongly influence diagnosis rates among a mixed neuropsychiatric population who have undergone detailed and highly consistent assessment for the symptoms that characterize delirium. The concordance between DSM-IV-defined cases and DSM-5 delirium in this dataset varied considerably (30% to 89%) according to the interpretation of DSM-5 criteria (strict versus relaxed), especially in relation to the requirements for acute onset, fluctuating course and role of disorientation. In the absence of convincing evidence to support a major change in the concept of delirium, as well as the desire to maintain the generalizability of existing literature to delirium knowledge, our data suggest that the more relaxed interpretation of the DSM-5 criteria should be used. The relaxed interpretation has considerable overlap with DSM-IV delirium, while also adding the benefit of additional precision to the definition that might allow for more focused research efforts. Importantly, this means ignoring the parenthetical notation to require simple disorientation to the environment in Criterion A, which potentially is duplicative of the ‘disorientation’ mentioned in Criterion C and inadequate from a phenomenological perspective to provide a surrogate for the complexity and breadth of what likely constitutes awareness of normal consciousness. The strict DSM-5 interpretation was clearly too restrictive when disorientation was also required in Criterion A, and too few patients were diagnosed as delirious. Conversely, another reason to favor the relaxed approach is that DSM-IV patients who were excluded by the relaxed DSM-5 criteria had significantly lower mean DRS-R98 scores below the DRS-R98 diagnostic cutoff score for delirium diagnosis.

The anchoring of awareness to simple testing of orientation in the Criterion A parenthesis is a significant departure from prior DSM versions and ICD, in which inattention is cardinal and an alteration of consciousness is left to the judgment of the observer who can incorporate a more full definition of awareness throughout the bedside interview. ‘Awareness’ presents a difficult concept to test in objective terms as it relates to the ability not only to accurately perceive and assimilate one’s surroundings but also to the appreciation and understanding of self. Recent work using functional magnetic resonance imaging (fMRI) in delirium [35] found alterations in the resting state default mode neural network, which reflects the quiet internal thinking mode. This reveals that the brain in a delirious person is not working normally and that, specifically, the internal thinking state – not just orientation to the external world - is impaired. Empirically, this is consistent with delirious patients’ thought process and comprehension impairments regarding self, others and situations. We strongly recommend that the guidance notes for DSM-5 advising impaired awareness is *‘manifested by a reduced orientation to the environment*’ not be followed in a strict sense. In addition, the use of ‘*and*’ means the identification of disorientation would become crucial to the diagnosis of delirium – a position that is not supported by studies that indicate a frequency that is not sufficiently high to justify a role as a mandatory diagnostic feature [23,36].

Another challenge relates to the optimal combination of the elements of criterion B since the DSM-5 text indicates that acute onset is ‘*usually’* evidenced by onset over hours or days, while disturbances ‘*tend’* to fluctuate over the course of the day. The implication is that neither description is mandatory, but that a pattern exists whereby either of these elements suffices reflected in the relaxed interpretation of this criterion. Using a strict DSM-5 approach (requiring both be present), this criterion accounted for almost three quarters of DSM-IV-diagnosed cases that were excluded even though the majority had mean DRS-R98 scores that exceeded the diagnostic cutoff score. Therefore, we do not recommend that both aspects of Criterion B be required in order to detect delirium. Similar findings have been described using the CAM algorithm where sensitivity is enhanced when *either* acute onset *or* symptom fluctuation (rather than acute onset *and* fluctuating symptoms) is required [37,38].

Criterion D addresses the attribution of symptoms to delirium versus other states, particularly dementia and newly introduced in DSM-5 is an exclusion from low arousal states ‘such as coma’ (although the guidance notes state that non-comatose patients giving even minimal responses to verbal stimulation should be classified as showing ‘severe inattention’). Operationalizing this aspect of diagnosis is challenging but can be achieved by applying tools with established discriminating capacity in cases complicated by comorbid dementia. For these analyses we used the DRS-R98 which distinguishes delirium from other neuropsychiatric conditions [22,29] and can thus help to clarify if a diagnosis of delirium should apply where there is evidence for a comorbid neuropsychiatric condition that may complicate clinical presentation.

Our finding of high concordance between clinically-determined DSM-IV diagnosis of delirium and the algorithm-generated DSM-5 relaxed diagnosis supports the usefulness of the DRS-R98 for identifying core diagnostic elements of delirium in an operationalized way. DSM criteria are aimed primarily towards clinicians and designed for use in everyday practice where flexibility and ‘common sense’ are desirable elements. However, they have also become the research standard and in this context require precise systematic methods for research use. The DRS-R98-based approach described herein overlapped substantially with the DSM-IV criteria identified by detailed clinical review by expert clinician-researchers and can thus assist where more systematic diagnosis is desired.

### Strengths and shortcomings

This is the first report that we are aware of that compares diagnosis using DSM-IV and DSM-5 by analyzing a large dataset using standardized assessments by carefully trained researchers conducted in a range of clinical settings where delirium is common. These methods are sufficiently operationalized to allow for precise application by expert assessors. A limitation of this study is that the DSM-IV diagnosed group underwent a clinical interview whereas the DSM-5 groups were diagnosed by *post hoc* application of DRS-R98 data to fulfill the criteria, and this may have led to some bias in the results, although we first ascertained high concordance between live and *post hoc* DSM-IV diagnoses. The population studied was derived from a mixture of referred and screened cases, such that the former are likely to have included a disproportionate number of patients with, for example, more florid presentations. We also included patients with other neurocognitive disorders (principally dementia) that pose common challenges to the accurate diagnosis of delirium. However, although the findings were similar across palliative care, psychiatry for later life and general hospital inpatients the pooled dataset does not include patients from some settings where delirium diagnosis is challenging due to issues of reduced arousal or where states such as stupor are especially relevant (for example, intensive care settings). Moreover, patients who were unable to co-operate with cognitive testing were not included in these studies so the relevance to patients with marked impairment of arousal requires further study [39].

## Conclusions

The concept of delirium described in DSM-5 overlaps considerably with DSM-IV delirium, but with narrower capture of delirium. Depending on the interpretation of criteria that is applied, between 11% and 70% of cases of DSM-IV delirium did not meet the new criteria, which has important implications for case identification in clinical and research activity. Overly-strict adherence for some new text details in DSM-5 criteria would greatly reduce the number of delirium cases diagnosed; however a more ‘relaxed’ approach renders DSM-5 criteria comparable to DSM-IV with minimal impact on their actual application. We also found that delirium diagnosis based upon relevant DRS-R98 items has substantial overlap with DSM-IV diagnoses from detailed clinical assessment, especially when interpreted that inattention is accompanied by impaired awareness flexibly defined in criterion A and that acute or subacute onset of symptoms with or without a fluctuating course accounts for criterion B of DSM-5. This relaxed interpretation of DSM-5 criteria has greater concordance with DSM-IV and is more inclusive of cases that have substantial delirium symptoms as measured on the DRS-R98. We, therefore, recommend this approach to delirium diagnosis according to DSM-5 criteria as it maintains the perceived strengths of DSM-IV in terms of simplicity and inclusiveness while clarifying how to address issues such as reduced alertness or inability to co-operate with assessments. Further studies can explore the therapeutic and prognostic relevance of different application of these criteria.

## Abbreviations

CAM: Confusion Assessment Method
CIRG: Cognitive Impairment Research Group
DRS-R98: Revised Delirium Rating Scale – 1998 version
DSM-5: *Diagnostic and Statistical Manual* fifth edition
DSM-IIIR: *Diagnostic and Statistical Manual* third edition revised
DSM-IV: *Diagnostic and Statistical Manual* fourth edition
fMRI: functional magnetic resonance imaging
ICD-10: *International Classification of Diseases* – Tenth Edition
IQCODE: Informant Questionnaire on Cognitive Decline in the Elderly
SPSS: statistical package for the social sciences
